# Validation of the Daily Spiritual Experience Scale Short Forms among Russian Orthodox Christian women

**DOI:** 10.1007/s10943-025-02402-7

**Published:** 2025-08-11

**Authors:** Fedor Shankov, Maria Böttche, Annett Lotzin

**Affiliations:** 1 Department of Psychiatry and Psychotherapy, University Medical Center Hamburg-Eppendorf, Hamburg, Germany; 2 Division of Clinical Psychological Intervention, Freie Universität Berlin, Berlin, Germany; 3 Institute for Clinical Psychology and Psychotherapy, MSH Medical School Hamburg, Hamburg, Germany

**Keywords:** Daily spiritual experience scale, Spirituality, Religiosity, Psychometric validation, Cultural adaptation, Russian Orthodox Christianity

## Abstract

Although widely used, the daily spiritual experience scale short form (S-DSES) has recently been criticized for conflating spiritual experiences with mental health outcomes, potentially obscuring true relationships between spirituality and health. This study evaluates the psychometric properties of three S-DSES versions in Russian Orthodox Christian women, addressing potential content-overlap concerns. A total of 1056 female participants primarily from Russia (92.9%), Belarus (4.5%), and Ukraine (2.5%) completed surveys at two time points (August 2023 and February 2024), with a subsample (n = 144) retested for reliability. Exploratory and confirmatory factor analyses supported a single-factor structure for all versions, which demonstrated strong internal consistency (Cronbach's alpha ranging from .80 to .87) and good test–retest reliability (intraclass correlation coefficients ranging from .75 to .81). The 7-item S-DSES demonstrated superior model fit compared to the original 6-item version. The 4-item theistic version, consisting of the items that reference God and show the least conceptual overlap with psychosocial constructs, retained significant, though weaker, correlations with mental health and well-being measures, indicating that the association between perceived divine closeness and psychological well-being persists beyond potential measurement overlap. The 7-item S-DSES exhibited the strongest psychometric properties and is recommended for comprehensive assessment of daily spiritual experiences in Russian Orthodox Christian women, whereas the 4-item theistic version offers a concise alternative for studies of health outcomes that require minimal overlap with psychosocial content.

**Supplementary Information:**

The online version contains supplementary material available at 10.1007/s10943-025-02402-7.

## Introduction

The association between spirituality, religiosity, and health has received substantial attention in recent decades. Systematic reviews and meta-analyses have shown that higher religiosity and spirituality (R/S) are generally positively associated with improved health and well-being (Koenig et al., 2024; Oman & Syme, 2018; Schwalm et al., 2022; Underwood & Vagnini, 2022; Yaden et al., 2022). Nevertheless, the literature frequently highlights methodological concerns: (1) ambiguous definitions of R/S constructs, (2) methodological challenges in operationalizing R/S with valid, reliable measures, and (3) limited validity testing of measures across diverse cultural contexts (Büssing, 2017; Herzog, 2020; Koenig et al., 2024; Richards et al., 2021).

Researchers emphasize distinguishing religiosity and spirituality as overlapping yet conceptually distinct constructs (Koenig et al., 2024). Religiosity refers to an organized system of beliefs, practices, rituals, social relationships, and symbols that are oriented toward a connection with the divine or transcendent, often practiced within a communal or institutional framework (Koenig et al.). In contrast, spirituality is conceptualized as a dynamic, personal pursuit involving experiences, beliefs, and practices aimed at establishing or nurturing a connection or union with the transcendent (de Brito Sena et al., 2021; Koenig et al.).

R/S measures often conflate spirituality with positive psychological outcomes, such as life satisfaction, personal growth, purpose, and peacefulness (Koenig & Carey, 2024). This conflation can obscure spirituality's unique contributions to mental health by confusing the construct with its presumed outcomes, potentially leading to erroneous conclusions. Widely used instruments like the Daily Spiritual Experience Scale require careful validation and methodological refinement to ensure that items measure R/S characteristics without inadvertently reflecting psychological well-being and mental health.

Furthermore, R/S constructs are highly culturally sensitive, requiring a careful and thorough approach to translation and validation of corresponding measures (Büssing, 2017; Koenig & Al Zaben, 2021; Richards et al., 2021). Most existing R/S measures and studies remain Western-centered, leaving a significant gap in understanding how R/S functions in other cultural and linguistic contexts (Herzog, 2020; Richards et al., 2021).

Russian-speaking populations illustrate this well: despite Russian being one of the most widely spoken languages globally, few R/S measures have undergone rigorous validation in this context. The resurgence of Orthodox Christianity following decades of Soviet-era atheism presents unique measurement challenges (Karpov et al., 2012). For instance, in the early 1980s, only 6–10% of Russians identified as religious, whereas by April 2024, a Levada Center poll found that 74% identified as Orthodox Christians. However, only 59% of them considered religion an important part of their daily lives, with women making up the majority. Despite this increase in religious identification, just 8% planned to attend church services, even for major celebrations like Orthodox Easter (Levada Center, 2024). This discrepancy suggests that for many, Orthodox Christianity functions more as a social identity than a source of personal faith and active religious practice. In this context, concepts such as "ethnodoxy" and "religious but not spiritual" may be particularly relevant (Karpov et al., 2012; Koenig & Carey, 2024, pp. 6–7). Methodologically sound studies examining the relationship between R/S and health in the Russian population remain underrepresented (Koenig et al., 2024).

In sum, research generally finds evidence that R/S are positively associated with mental health and serve as resources for coping and resilience in stressful times. However, these findings are complicated by unclear R/S definitions, measurement challenges, and limited cross-cultural validation. This study aims to address these challenges by validating a questionnaire that captures the experiential dimension of both religiosity and spirituality, is economically feasible, and has been widely studied in the context of health and spirituality research but not within Russian-speaking populations: the Daily Spiritual Experience Scale Short Form (S-DSES; Idler et al., 2003; Underwood, 2011; Underwood & Teresi, 2002; Underwood & Vagnini, 2022).

This study seeks to contribute to a more nuanced understanding of R/S and its relationship with health in Russian-speaking populations by psychometrically testing modified, culturally, conceptually, and linguistically adapted Russian versions of the S-DSES among Orthodox Christian women.

## Method

### Study Design

This cross-sectional validation study was conducted at two distinct time points (August 2023 and February 2024). Three versions of the Daily Spiritual Experience Scale Short Form (S-DSES) were validated: (1) a 6-item version similar to the original S-DSES, (2) a 7-item version including an additional closeness-to-God item, and (3) a 4-item theistic version that omits items most prone to overlap with mental health constructs, as recommended by Koenig and Carey (2025). The study comprised two phases: (a) translation and cultural adaptation and (b) psychometric evaluation of the Russian S-DSES versions. Procedures adhered to guidelines for translation and validation of scales (Koenig & Al Zaben, 2021) and followed the Consensus-Based Checklist for Reporting of Survey Studies (CROSS; Sharma et al., 2021).

### Study Sample

A total of 1,056 Russian Orthodox Christian women participated in this study, ranging in age from 18 to 77 years (M = 45.17, SD = 10.11). Based on IP location data collected through the 1KA.si platform, participants predominantly resided in Russia (92.9%), with smaller representations from Belarus (4.5%), Ukraine (2.5%), and Serbia (0.5%). The remaining participants (< 1%) were distributed across other countries. Among Russian participants, 38.6% were from Moscow and 14.7% from St. Petersburg, with others distributed across various urban and rural regions. It should be noted that IP-based geolocation represents an estimation method with inherent limitations but was the most appropriate approach for determining participant location while maintaining anonymity in this online survey.

The majority of participants (80.7%) reported having completed higher education (60.4% with a specialist degree or other higher education qualification, 17.0% multiple degrees, and 3.3% doctoral degrees). Most participants were employed (74.1%; 37.2% full-time, 37.0% part-time), and over half (53.9%) were married. Regarding family structure, 51.3% had one or two children, 17.4% had three or more children, and 31.3% had no children. In terms of socioeconomic status, 44.1% self-identified as upper-middle class, while 28.2% identified as lower-middle class. Comprehensive demographic characteristics for the total sample and subsamples are presented in Table 1.

**Table 1:** Demographic characteristics of study samples

	Total Sample (*N* = 1056)		Subsample 1 (*n* = 530)		Subsample 2 (*n* = 526)		Subsample 2.1 (*n* = 144)	
	*M*	*SD*	*M*	*SD*	*M*	*SD*	*M*	*SD*
Age	45.17	10.11	44.50	10.57	45.85	9.61	45.77	8.90

	*n*	%	*n*	%	*n*	%	*n*	%
Education Level								
Basic education	5	0.47	2	0.38	3	0.57	1	0.69
General secondary	23	2.17	14	2.64	9	1.71	0	0
Vocational second	126	11.91	63	11.89	63	11.98	19	13.19
Incomplete higher	50	4.73	29	5.47	21	3.99	4	2.78
Higher education	639	60.40	314	59.24	324	61.60	79	54.86
Multiple higher	180	17.01	92	17.36	87	16.54	34	23.61
Doctorate	35	3.31	16	3.02	19	3.61	7	4.86
Marital Status								
Married	570	53.87	283	53.40	286	54.37	83	57.64
Divorced	188	17.77	95	17.92	92	17.49	20	13.89
Never married	228	21.55	116	21.89	112	21.29	33	22.92
Widowed	51	4.82	24	4.53	27	5.13	6	4.17
Cohabiting	21	1.98	12	2.26	9	1.71	2	1.39
Child Status								
No children	331	31.28	165	31.13	166	31.56	43	29.86
One/two children	543	51.32	269	50.75	272	51.71	76	52.78
More than three children	184	17.39	96	18.11	88	16.73	25	17.36
Employment Status								
Full-time	393	37.15	201	37.92	192	36.50	48	33.33
Part-time	391	36.96	199	37.55	191	36.31	53	36.81
Unemployed, seeking	78	7.37	37	6.98	40	7.60	11	7.64
Not in labor force	114	10.77	51	9.62	63	11.98	20	13.89
Retired	67	6.33	35	6.60	32	6.08	9	6.25
Disabled, not working	15	1.42	7	1.32	8	1.52	3	2.08
Socioeconomic Status								
Extreme poverty	28	2.65	17	3.21	11	2.09	2	1.39
Low income	188	17.77	89	16.79	98	18.63	20	13.89
Lower-middle	298	28.17	159	30.00	139	26.43	39	27.08
Upper-middle	467	44.14	223	42.07	243	46.20	72	50.00
High income	77	7.28	42	7.93	35	6.65	11	7.64

*M* mean, *SD* standard deviation; *n* number of participants

The survey initially received 3,051 visitors, of whom 2,371 (77.7%) proceeded to the first page, and 1469 (48.14%) fully completed the survey. Following data cleaning (see “Data Cleaning” section), *n* = 413 participants were excluded, resulting in a final sample of *N* = 1056. The number of male participants was insufficient for meaningful statistical analysis, making their inclusion unrepresentative. As a result, this study focused exclusively on women, and findings should not be generalized to male Orthodox populations (see “Limitations” section for further discussion).

The total sample was divided into subsamples based on the time of two measurements to facilitate factor analyses and test–retest reliability assessment. Subsample 1 (August 2023) included *n* = 530 participants. Subsample 2 (February 2024) included *n* = 526 participants who did not overlapwithSubsample 1. Subsample 2.1 (*n* = 144) included participants who completed both time points. Data from these n = 144 participants were ex cludedfromtheEFA(inSubsample 1) but included in the CFA (Subsample 2); data from both measurement points were used to assess test–retest reliability.

### Study Procedure

Participants were recruited through interrelated public Telegram channels affiliated with the “Psychology for Church” and “Psychology with Marina Filonik” educational projects, which focus on psychological counseling training and psychoeducation for Russian Orthodox Christian women. Ethical approval for the study was obtained from the Ethics Committee for Psychological and Pedagogical Research at the Institute of Pedagogy and Psychology of Education, Moscow City Pedagogical University. Participants provided informed consent before starting the survey and completed an online survey hosted on the 1KA platform (Centre for Social Informatics, Faculty of Social Sciences, University of Ljubljana; https://www.1ka.si). The survey was publicly accessible, and its functionality was tested prior to data collection. The majority of items were mandatory, with reminders prompting completion to ensure accurate calculations and the provision of individual results to participants.

After completion, participants received a summary of their individual scores along with general population norms for validated measures, definitions of measured constructs, and self-help practices for improving mental health and mindfulness. These personalized results served as an incentive for participation but were provided only after all measures were completed, thus not influencing response patterns.

To detect potential duplicate submissions and to enable linking participants who completed both time points, anonymized IDs were generated by combining non-identifying personal details (i.e., birth date day number, shoe size digits, and the first letters of the mother's name). The median completion time was 27 min and 57 s.

### Measures

#### Translation, Cultural Adaptation of the Daily Spiritual Experience Scale Short Form

The original S-DSES consists of six items and was developed as part of the “National Institute on Aging/Fetzer Institute Short Form for the Measurement of Religiousness and Spirituality” used in the General Social Survey (Idler et al., 2003). The scale was designed to assess an individual’s perception of a regular spiritual connection with the transcendent (e.g., God, Nature), focusing on daily spiritual experience rather than extraordinary events, specific spiritual beliefs, or religious behaviors; is deliberately independent of institutional or organizational religious activity and was differentiated from other psychosocial constructs during its development (Underwood, 2006; Underwood & Teresi, 2002). Notably, the S-DSES was not established psychometrically from the full DSES but rather developed ad hoc, with items 2 and 4 resulting in a merge of four items from the full DSES form (Underwood, 2011, p. 36). Despite this, the 6-item scale has been found to be highly correlated with the longer version with similar effects on selected variables in large independent samples (Ellison & Fan, 2008). The S-DSES has been widely used and validated across various cultural contexts with consistently strong psychometric properties (e.g., Bailly & Roussiau, 2010; Loustalot et al., 2011).

Respondents rate each item on a six-point Likert scale, with response options ranging from 1 (never or almost never) to 6 (many times a day). The total score of the S-DSES is calculated as the mean of all items, resulting in a range from 1 to 6. Higher scores indicate a greater frequency of spiritual experience in the respondent's life. The original S-DSES showed good internal consistency (*α* = .91) in a sample of *N* = 1,374 US American adults (Idler et al., 2003). The interclass correlation coefficient for the 6-item short-form was .73 (*n* = 50; Underwood & Teresi, 2002).

Following permission from the original DSES author, the provided Russian translation was culturally adapted in accordance with established guidelines (Koenig & Al Zaben, 2021). The key adaptations to the original S-DSES included instructions guided by the full DSES version, allowing participants to substitute “God” with a personally meaningful divine concept and the addition of “spirituality” to the strength and comfort item to produce more agreement with the original scale items. In addition, in line with the author’s recommendation (Underwood, 2011), a seventh item was introduced to assess closeness to God, calibrating the existing item “I desire to be closer to or in union with God.”

First, a preliminary version was pilot-tested with 20 male Russian Orthodox clergy professionals attending psychiatry training at the Mental Health Research Center in Moscow. Their feedback indicated a need to align specific terms with Orthodox ascetic traditions that discourage emotional expressions of spirituality (Naumescu, 2012). Based on these insights, the translation team—comprising of two bilingual researchers, a professional translator, a priest, and a psychiatrist—reviewed each item for linguistic and conceptual accuracy. The term “feeling” in two items was replaced with “perezhivanie,” which more closely conveys “experiencing” in Russian (Vasilyuk, 1991, p. 9); and the word “comfort,” prone to calque distortions, was changed to “uteshenie” (“solace”). Next, the revised version was piloted on 10 voluntary respondents. All of them reported no difficulties in understanding the items. Completion of the 7-item version required approximately 1–2.5 min. Finally, the back-translation and all modifications were approved by the original DSES author. The resulting 7-item Russian S-DSES, alongside the original English wording, is available in Supplementary Material Table S1.

The 4-item theistic version was formed by omitting the items that refer to R/S strength and solace, deep inner peace or harmony, and being spiritually touched by beauty (Items 2, 3, and 6), in accordance with Koenig and Carey’s (2025) recommendation to exclude content that may tap mental health constructs rather than religiosity/spirituality; all remaining items explicitly reference God, forming a concise theistic subset suitable for health and clinical research.

Therefore, this study examines the psychometric properties of three S-DSES versions: (1) an adapted 6-item S-DSES reflecting the original scale, (2) a 7-item version incorporating the item “How close do you feel to God?”, and (3) a 4-item theistic version.

#### The Centrality of Religiosity Scale (CRS)

The *Centrality of Religiosity Scale* (CRS; Huber, 2003, 2012) assesses the centrality, importance, and salience of religiosity within an individual’s life. Grounded in Allport and Ross’s psychological model of religiosity and Glock and Stark’s multidimensional model of religion, the CRS explores how core religious beliefs shape daily practices, attitudes, and values. The Russian version of the short CRS was validated by Ackert et al. (2020), demonstrating strong internal consistency (*α* = .85) and time-invariance across two large-scale surveys conducted in 2008 (*N* = 894) and 2019 (*N* = 1768). The full CRS implemented in this study consists of 15 items grouped into five three-item subscales—Public Practice, Private Practice, Religious Experience, Ideology, and Intellectual Engagement. Respondents use a series of scales—5-point for importance and 5-, 6-, and 8-point scales for frequency—and standardized to a 1–5 range. For instance, a respondent might be asked, “How often do you experience situations in which you feel that God or something divine intervenes in your life?” and respond from 1 (never) to 5 (very often). The total CRS score is the mean of all 15 items, ranging from 1 to 5, with higher scores indicating greater centrality of religiosity.

In this study, the CRS total score demonstrated good internal consistency (Cronbach’s *α* = .86). Subscale reliability estimates ranged from acceptable to good (Intellectual: *α* = .73; Ideological: *α* = .77; Public Practice: *α* = .77; Private Practice: *α* = .65; Experience: *α* = .80).

#### Mental Health and Well-being Measures

This study included measures frequently employed in prior DSES research to assess criterion-related validity (Underwood & Vagnini, 2022). These measures were selected to capture constructs empirically linked to daily spiritual experience, such as mental health and overall well-being, and to facilitate meaningful comparisons with previous validation studies.

*Depression Anxiety Stress Scale* (DASS-21). The DASS-21 (Lovibond & Lovibond, 1995) is composed of three 7-item subscales assessing depression, anxiety, and stress. Items are rated on a 4-point scale ranging from 0 (never) to 3 (always). The Russian version was validated in a sample of *N* = 1,153 participants, reporting Cronbach’s *α* values of .90, .85, and .91 for the depression, anxiety, and stress subscales, respectively, and .95 for the overall distress index (Zolotareva, 2021). In the current study, internal consistency remained good, with *α* = .81 for anxiety, *α* = .87 for depression, *α* = .88 for stress, and *α* = .94 for the total score.

*Satisfaction With Life Scale* (SWLS). The SWLS (Diener et al., 1985) comprises five items rated on a 7-point scale from 1 (strongly disagree) to 7 (strongly agree), producing a single total score. Higher scores reflect greater life satisfaction. The Russian version showed good internal consistency (*α* = .82) and test–retest stability (*r* = .70 over two months) in a sample of *N* = 7,091 participants (Osin & Leontiev, 2020). In the present study, the SWLS demonstrated acceptable internal consistency (*α* = .79).

*Self-Rated Health* (SRH). SRH (Ware & Sherbourne, 1992) is a single-item measure asking participants to rate their overall health on a 5-point scale from 1 (very bad) to 5 (excellent). SRH has been identified as a robust predictor of disability, morbidity, and mortality, including within Russian populations studied by the World Health Organization (Nicholson et al., 2005).

*Mindful Attention Awareness Scale* (MAAS). The MAAS (Brown & Ryan, 2003) is a 15-item, 6-point scale designed to measure trait mindfulness, focusing on the individual’s capacity for present-moment attention and awareness. The Russian validation, conducted on two independent student samples (*N* = 531), indicated good internal consistency for the total score (*α* = .79) and the deautomatization subscale (*α* = .83), while other subscales (concentration and attention) were not retained due to poor reliability (Mitina et al., 2021). In this study, both the general factor and deautomatization subscale exhibited good internal consistency (*α* = .87 and .89, respectively).

### Data Analyses

#### Data Cleaning

Data cleaning was performed using Excel and involved identifying and removing duplicate entries, outliers with unusually low CRS and high DASS-21 scores, and patterned responses (i.e., identical answers to consecutive items across multiple scales within an implausibly short completion time), which indicate potential data quality issues. During this process, *n* = 413 participants were excluded.

#### Statistical Software

All analyses were conducted using JASP (JASP Team, 2024). Descriptive statistics were followed by exploratory and confirmatory factor analyses, reliability assessments, and validity testing. Normality was evaluated using the Kolmogorov–Smirnov test, with nonparametric methods applied where assumptions were violated.

#### Descriptive Statistics

Frequencies, means, and standard deviations (*SD*) were calculated for participant demographics and primary study variables. This provided an overview of the sample’s characteristics and the distribution of responses on the S-DSES and related measures.

#### Validity and Reliability Assessment

*Structural Validity*. Exploratory Factor Analysis (EFA) was conducted on Subsample 1 (*n* = 530) using direct oblimin rotation to identify the factor structure. Confirmatory Factor Analysis (CFA) was then performed on Subsample 2 (*n* = 526) to verify the factor structure identified by the Exploratory Factor Analysis (EFA). Model fit was evaluated using the Chi-Square Statistic (*χ*^*2*^), Chi-Square to Degrees of Freedom Ratio (*χ*^*2*^*/df*), Root Mean Square Error of Approximation (RMSEA), Standardized Root Mean Square Residual (SRMR), Comparative Fit Index (CFI), and Tucker–Lewis Index (TLI). Acceptable model fit criteria were defined as *χ*^*2*^*/df* < 3.00, RMSEA < .08, SRMR < .10, and CFI/TLI > .90, while good fit criteria were stricter: *χ*^*2*^*/df* < 2.00, RMSEA < .06, SRMR < .05, and CFI/TLI > .95 (Harrington, 2009).

*Internal Consistency and Test–Retest Reliability*. Cronbach’s *α* was used to assess internal consistency (acceptable range .70-.95; Tavakol & Dennick, 2011). For test–retest reliability assessment, the intraclass correlation coefficient (ICC) was calculated for Subsample 2.1 (*n* = 144) over a six-month interval. ICC values ≥ .60 indicate good and ≥ .75 excellent test–retest reliability (Kurtz, 2017).

*Convergent Validity*. The Kolmogorov–Smirnov test assessed data normality. Nonparametric Spearman’s rank correlation (*ρ*) examined associations between S-DSES versions and the CRS total and religious experience subscale. AVE (Average Variance Extracted) was also considered, with AVE > .50 indicating good convergent validity (Fornell & Larcker, 1981).

*Criterion-related Validity*. Spearman’s ρ examined relationships between S-DSES scores and measures of mental health (DASS-21), life satisfaction (SWLS), self-rated health (SRH), and mindfulness (MAAS). Consistent with prior research (Underwood & Vagnini, 2022), we hypothesized negative correlations with depression and stress and positive correlations with life satisfaction, self-rated health, and mindfulness. All *p* values are reported as two-tailed, and effect sizes are presented as appropriate.

## Results

### Descriptive Statistics

Table 2 summarizes descriptive statistics for the S-DSES versions. The mean total scores were 3.80 (SD = 0.91) for the 6-item version, 3.81 (SD = 0.92) for the 7-item version, and 3.77 (SD = 1.02) for the 4-item version. Although the Kolmogorov–Smirnov test indicated non-normality across all versions, visual inspection suggested an approximate normal distribution.

**Table 2:** Mean and standard deviation of the S-DSES items

Shortened item wording	*M*	*SD*
1. God’s presence	3.48	1.29
2. Strength in R/S	4.04	1.18
3. Deep inner peace/harmony	3.05	1.05
4. Desires to be in union	4.01	1.17
5. Love directly/through others	3.73	1.21
6. Touched by beauty	4.47	1.16
7. Close	3.85	1.47
Total score 4-item version	3.77	1.02
Total score 6-item version	3.80	0.91
Total score 7-item version	3.81	0.92

*N* = 1056,* M* mean, *SD* standard deviation

### Validity and Reliability Assessment

#### Structural Validity

*Exploratory Factor Analysis (EFA)*. Conducted with Subsample 1 (n = 530), EFA identified a one-factor structure across all versions. Factor loadings exceeded .60 for all items in all versions (Table 3), with Item 2 (“I find strength and solace in my religion or spirituality”) showing the highest loading (.84), while Item 6 (“I am spiritually touched by the beauty of creation”) had the lowest (.61) in 6- and 7-item versions.

**Table 3:** Factor loadings for S-DSES items

Shortened item wording	6-item version factor loadings	Uniqueness	7-item version factor loadings	Uniqueness	4-item version factor loadings	Uniqueness
1. God’s presence	.74	.46	.74	.45	.74	.44
2. Strength in R/S	.84	.29	.84	.30	-	-
3. Deep inner peace	.69	.52	.70	.51	-	-
4. Desires to be in union	.72	.47	.72	.47	.62	.62
5. Love directly / others	.78	.38	.79	.38	.80	.37
6. Touched by beauty	.62	.62	.61	.62		
7. Close	-	-	.66	.57	.63	.60

Subsample 1, *n* = 530

*Confirmatory Factor Analysis (CFA)*. CFA was performed on Subsample 2 (*n* = 526) to validate the one-factor structure identified through EFA. Table 4 summarizes the fit indices for the 6-item, 7-item, and 4-item versions. All three models met the criteria for acceptable fit thresholds. Among these, the 4-item version demonstrated the strongest overall model fit, followed by the 7-item and 6-item versions.

**Table 4:** Global goodness of fit of the tested S-DSES versions

	*χ2*	*df*	*p*	*χ2/df*	RMSEA	SRMR	TLI	CFI
Acceptable fit values*				< 3.00	< .08	< .10	> .90	> .90
Good fit values*				< 2.00	< .06	< .05	> .95	> .95
6-item version	28.20	9	.00	3.13	.06	.02	.99	1.00
7-item version	36.42	14	.00	2.60	.05	.02	.99	1.00
4-item version	0.27	2	.87	0.14	.00	.00	1.00	1.00

Subsample 2, *n* = 526

*χ2/df* ratio of the chi-square statistic to the respective degrees of freedom, *RMSEA* root mean square error of approximation, *SRMR Standardized Root Mean Residual, TLI* Tucker–Lewis index, *CFI* comparative fit index

^*^Harrington (2009)

#### Internal Consistency

Cronbach’s alpha was calculated based on the entire sample data (*N* = 1056). The results indicated good reliability for all versions: .87 for the 7-item version, .87 for the 6-item version, and .80 for the 4-item version.

#### Test–Retest Reliability

Test–retest reliability was assessed using Subsample 2.1 (*n* = 144) over a six-month interval. The Intraclass Correlation Coefficients (ICC) indicated excellent reliability, with values of .81 for the 6-item version, .80 for the 7-item version, and .75 for the 4-item version.

#### Convergent Validity

Convergent validity was assessed by examining correlations between the S-DSES versions, the CRS total score, and its Religious Experience subscale. The Kolmogorov–Smirnov test indicated non-normality for both scales, warranting the use of nonparametric Spearman correlations. All three S-DSES versions demonstrated significant positive correlations with the included measures, with Spearman’s *Rho* values exceeding .64 for both the CRS total score and the CRS Religious Experience subscale. Additionally, the Average Variance Extracted (AVE) scores were calculated as follows: .54 for the 6-item version, .53 for the 7-item version, and .53 for the 4-item version. Detailed results of the Spearman’s *Rho* for the tested S-DSES versions and key variables are provided in Supplementary Materials Table S2.

#### Criterion-related Validity

Criterion-related validity was examined by analyzing the relationships between the S-DSES versions and theoretically related variables, including depression, anxiety, stress, life satisfaction, self-rated health, and mindfulness (for Spearman correlation coefficients, see Table S2 in Supplementary Materials). All versions exhibited significant relationships with these measures, with varying effect sizes across constructs and S-DSES versions; however, the 4-item version exhibited reduced correlations with mental health and well-being measures.

## Discussion

The primary objective of this study was to psychometrically evaluate three Russian-language versions of the Daily Spiritual Experience Scale Short Form (S-DSES) among Russian Orthodox Christian women. We assessed the psychometric properties of (1) the adapted original 6-item S-DSES version, (2) an expanded 7-item S-DSES version incorporating a closeness-to-God item, and (3) a 4-item theistic version, which excludes items potentially conflated with mental health outcomes.

### Interpretation of Key Findings

The results of the structural validity analysis supported a unidimensional structure across all three versions of the Russian S-DSES, consistent with prior validation studies (Underwood & Teresi, 2002; Underwood & Vagnini, 2022). Each version demonstrated high internal consistency and good test–retest reliability, reinforcing the notion that daily spiritual experience may function as a relatively stable trait, particularly among individuals engaged in structured religious practice. Notably, adding the 'closeness to God' item in the 7-item version slightly enhanced model fit, as reflected in improved χ^2^/df and RMSEA indices. This finding aligns with the original DSES author's argument for including this item (Underwood, 2011).

All three versions exhibited significant positive correlations with the Centrality of Religiosity Scale (CRS), particularly its Religious Experience subscale, providing strong support for their convergent validity. Consistent with prior research, S-DSES scores were more strongly associated with private religious practice than with public religious behaviors (Ellison & Fan, 2008), reinforcing the scale’s focus on personal spiritual experience rather than communal or institutional religiosity.

All three versions correlated significantly with mental health and well-being measures, aligning with previous findings (Underwood & Vagnini, 2022) and supporting their criterion-related validity. As expected, the 4-item theistic version demonstrated weaker associations with mental health-related constructs, including the absence of a significant correlation with the DASS-21 Anxiety subscale. Despite these attenuated correlations, the 4-item version maintained significant associations with depression, life satisfaction, and mindfulness. This pattern suggests that experiences of divine connection meaningfully contribute to the established relationship between spirituality and mental health.

The mean scores of the Russian S-DSES items aligned with prior data from the General Social Survey (GSS; Fetzer, 2003; Ellison & Fan, 2007), except for the item “Deep inner peace/harmony,” which demonstrated significantly lower scores and reduced variability in the current sample compared to the GSS. These differences may reflect the likely influence of the sociopolitical context during data collection, particularly the ongoing Russo-Ukrainian war, which could have influenced participants’ perceptions of peace and well-being. This highlights the contextual sensitivity of the S-DSES and underscores its cross-cultural applicability.

Some previous studies, particularly those involving both religious and nonreligious populations, have reported a two-factor DSES structure distinguishing between “theistic” and “non-theistic (self-transcendent)” items (Hammer & Cragun, 2019). However, the 4-item theistic version suggests an alternative interpretation, wherein the division may reflect a distinction between spiritual connection and its experiential outcomes. This perspective aligns with a recent proposal by Koenig and Carey (2025) to analyze these experiential mental health-related items as a separate mediator in the relationship between R/S items with less potential overlap with mental health constructs and health in similar R/S measures.

### Theoretical and Practical Implications

This study contributes to the growing body of research on the psychometric evaluation of R/S measures, specifically addressing an understudied population of Russian Orthodox Christians. Theoretically, our findings support the conceptualization of spirituality as a distinct experiential construct, albeit one that frequently coexists with psychological well-being (Koenig & Carey, 2024; Oman & Syme, 2018). The validation of multiple S-DSES versions provides researchers studying Russian-speaking populations with reliable options for measuring spiritual experiences. Furthermore, the patterns of association between these versions and mental health measures offer insight into the robustness of spirituality–well-being relationships, even after potentially confounding item content is removed.

Cultural sensitivity remains crucial in R/S measurement, as historical, social, and religious contexts shape both the expression of spirituality and the interpretation of scale items (Büssing, 2017; Koenig & Al Zaben, 2021; Richards et al., 2021). The culturally adapted Russian versions of the S-DSES developed in this study provide more accurate assessments that can facilitate evidence-based, culturally sensitive interventions and inform practitioners working within Orthodox Christian contexts (Koenig et al., 2024; Underwood & Vagnini, 2022).

### Limitations

Although this study has several strengths, including robust sample size, distinct subsamples for exploratory and confirmatory factor analysis, and adherence to established validation protocols, several limitations should be acknowledged.

First, participants were self-selected Russian-speaking Orthodox Christian women recruited through interconnected online platforms devoted to Orthodox Christianity and psychology. This gender-specific, web-based sampling constrains representativeness and prevents generalization to men or non-Orthodox populations. Future studies should incorporate more diverse samples and broader recruitment strategies, including offline data collection and community-based sampling.

Second, all variables were self-reported, leaving data susceptible to social-desirability bias and differences in interpreting spiritual language. Employing observational and behavioral indices, qualitative interviews, and implicit measures could mitigate mono-method bias and enhance construct validity. Longitudinal and experimental designs are needed to clarify temporal and causal relations between daily spiritual experience and mental health outcomes.

Third, the S-DSES disproportionately reflects items that load more heavily on constructs such as positive affect (e.g., peace, strength), which may confound associations with mental health. Future studies should adapt and validate the full DSES, which offers broader content coverage and reduces psychological overlap (Koenig & Carey, 2024; Underwood, 2011).

## Conclusion

This study provides psychometric validation of Russian S-DSES versions, offering researchers culturally adapted measures for assessing daily spiritual experience and advancing R/S research in Russian-speaking populations. The 7-item version demonstrates strong reliability and validity, making it a robust tool for spirituality assessments and cross-cultural comparisons. Meanwhile, the 4-item theistic version serves as a methodologically rigorous alternative, particularly suited for R/S and health research, where minimizing conceptual overlap with mental health constructs is essential.

## Supplementary Information

Below is the link to the electronic supplementary material.

Supplementary file1 (DOCX 22 kb)

## Data Availability

Data supporting the findings of this study are available from the corresponding author upon reasonable request.
