# Supplementary material for: Validation of the Daily Spiritual Experience Scale Short Forms among Russian Orthodox Christian women
Source: J Relig Health. 2025 Aug 11;65(1):794–811. doi: 10.1007/s10943-025-02402-7 (PMC12913258; doi:10.1007/s10943-025-02402-7)
Supplement: Supplementary file 1 — Supplementary file1 (DOCX 22 kb) [file 10943_2025_2402_MOESM1_ESM.docx]

# Supplementary Materials

## Table S1

*Russian Daily Spiritual Experience Scale Short Form*

| Оцените, как часто вы это непосредственно переживаете* *(How often do you experience the following*?)*:  *** Во многих утверждениях употребляется слово «Бог». Если вас смущает это слово, пожалуйста, подразумевайте под ним что-то другое, что вызывает у вас мысли о высшем или священном для вас *(* A number of items use the word God. If this word is not a comfortable one for you, please substitute it with another word that calls to mind the divine or holy for you).* | | | | | | | | | |
| --- | --- | --- | --- | --- | --- | --- | --- | --- | --- |
|  | **Никогда**  (Never) | **Изредка**  *(Once in a while)* | | **В некото-рые дни**  *(Some days)* | **Большин-cтво дней**  *(Most days)* | | **Ежедне-вно**  *(Every day)* | | **Ежедне-вно многокра-тно**  *(Many times a day)* |
| 1. Я переживаю присутствие Бога *(I feel God’s presence)*. | 1 | 2 | | 3 | 4 | | 5 | | 6 |
| 2. Я обретаю силу и утешение в моей вере или духовности *(I find strength and comfort in my religion).* | 1 | 2 | | 3 | 4 | | 5 | | 6 |
| 3. Я чувствую глубокое внутреннее умиротворение или гармонию *(I feel deep inner peace or harmony).* | 1 | 2 | | 3 | 4 | | 5 | | 6 |
| 4. Я стремлюсь быть ближе к Богу или в единении с Ним *(I desire to be closer to or in union with God).* | 1 | 2 | | 3 | 4 | | 5 | | 6 |
| 5. Я переживаю любовь Бога ко мне напрямую или через других людей *(I feel God’s love for me, directly or through others).* | 1 | 2 | | 3 | 4 | | 5 | | 6 |
| 6. Меня духовно трогает красота творения *(I am spiritually touched by the beauty of creation).* | 1 | 2 | | 3 | 4 | | 5 | | 6 |
| 7. В целом, насколько вы переживаете близость к Богу *(How close do you feel to God)*? | 1 = **Совсем не близок(ка)**  *(Not close)* | | 3 = **Несколько близок(ка)**  *(somewhat close)* | | | 5 = **Очень близок(ка)**  *(very close)* | | 6 = **Близок(ка) насколько это возможно**  *(as close as possible)* | |

© Lynn Underwood [www.dsescale.org](http://www.dsescale.org) permission required to copy or publish

**Обработка данных (Data Processing).** Для оценки выраженности повседневных духовных переживаний рекомендуется вычислить среднее арифметическое баллов по всем пунктам. Для исследований в области здоровья и благополучия, рекомендуется исключить пункты 2, 3, 6 из обработки *(To assess the level of daily spiritual experience, it is recommended to calculate the average of the scores across all items. For health and well-being studies, it is recommended to exclude Items 2, 3, and 6 from the analysis).*

| Table S2 *Spearman's Rank Correlation Coefficient for the Tested S-DSES Versions and Key Variables* | | | |
| --- | --- | --- | --- |
| Variable | 6-item version | 7-item version | 4-item version |
| CRS | .65*** (.62, .69) | .65*** (.62, .69) | .64*** (.61, .68) |
| Religious experience | .64*** (.60, .67) | .65*** (.61, .68) | .65*** (.62, .69) |
| Public practice | .35*** (.30, .41) | .35*** (.30, .40) | .34*** (.29, .40) |
| Private practice | .52*** (.47, .56) | .52*** (.47, .56) | .51*** (.46, .55) |
| Ideology | .39*** (.34, .44) | .40*** (.34, .45) | .40*** (.35, .45) |
| Intellectual | .42*** (.36, .46) | .40*** (.35, .45) | .38*** (.33, .43) |
| SWLS | .34*** (.29, .40) | .35*** (.29, .40) | .32*** (.26, .37) |
| DASS-21 | -.27*** (-.33, -.22) | -.27*** (-.32, -.21) | -.22*** (-.27, -.16) |
| Depression | -.37*** (-.42, -.31) | -.36*** (-.41, -.31) | -.31*** (-.36, -.26) |
| Anxiety | -.09** (-.15, -.03) | -.08** (-.14, -.02) | -.05 (-.11, .02) |
| Stress | -.22*** (-.27, -.16) | -.22*** (-.27, -.16) | -.17*** (-.23, -.11) |
| Self-Rated Health | .18*** (.12, .24) | .18*** (.12, .24) | .16*** (.10, .21) |
| MAAS | .24*** (.19, .30) | .25*** (.20, .31) | .24*** (.18, .30) |
| Deautomatization | .20*** (.15, .26) | .22*** (.16, .27) | .21*** (.15, .26) |
| Demographic Characteristics |  |  |  |
| Age | .06* (.00, .12) | .07* (.01, .13) | .05 (-.01, .11) |
| Education level | .10** (.04, .16) | .09** (.03, .15) | .08** (.02, .14) |
| Child status | .06* (.00, .12) | .08* (.02, .14) | .08** (-.02, .14) |
| Socioeconomic status | .10** (.04, .16) | .11*** (.05, .17) | .11*** (.05, .16) |
| *Note*. *N* = 1,056. 95% Confidence Intervals in parentheses. CRS = Centrality of Religiosity Scale; SWLS = Satisfaction with Life Scale; DASS-21 = Depression Anxiety Stress Scale; MAAS = Mindful Attention Awareness Scale. * *p* < .05, ** *p* < .01, *** *p* < .001. | | | |
